# Supplementary material for: Neutrophil extracellular traps released by neutrophils impair revascularization and vascular remodeling after stroke
Source: Nat Commun. 2020 May 19;11:2488. doi: 10.1038/s41467-020-16191-y (PMC7237502; doi:10.1038/s41467-020-16191-y)
Supplement: Supplementary file 1 — Supplementary Information [file 41467_2020_16191_MOESM1_ESM.pdf]

Neutrophil extracellular traps released by neutrophils impair revascularization and vascular remodeling after stroke

Kang et al.

Supplementary Figures

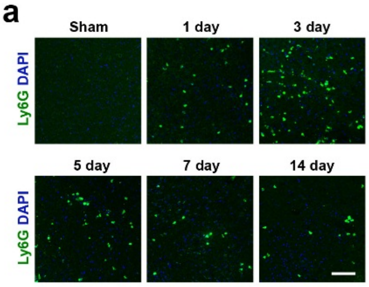

Supplementary Figure 1. Neutrophils accumulated inside brain vessels and the parenchyma during all stages of stroke. a Representative images of Ly6G-positive neutrophils in the peri-infarct cortex of mice subjected to stroke, compared to sham-operated mice. Bar = 40 μm. Independent experiments are repeated at least three times.

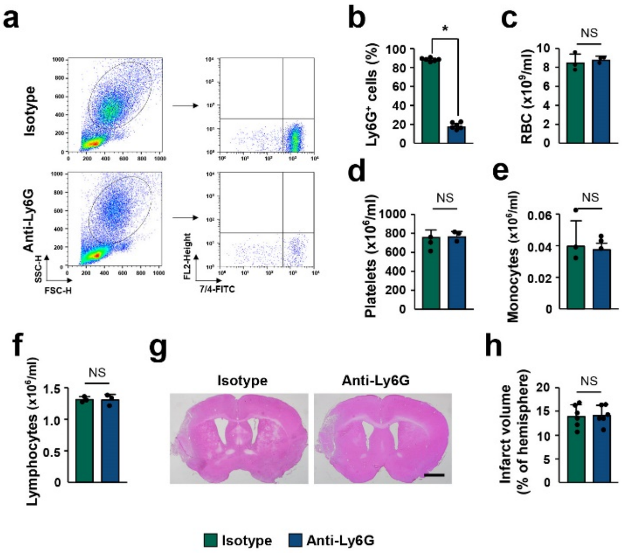

Supplementary Figure 2. Effects of neutrophil depletion on the number of blood neutrophils, other blood cells, and infarct volumes. a, b Representative flow cytometry analysis (a) and quantification (b) of neutrophils in peripheral blood at 14 days after stroke. For gating , granulocytes were gated using forward scatter height (FSC-H) versus side scatter height (SSC-H), neutrophil surface marker positive events were gated using a FITC-conjugated rat monoclonal anti-mouse neutrophil antibody (anti-7/4, 1:300, Abcam, cat. no.

ab53453) versus fluorescence height (n = 6), unpaired two-tailed Student’s t test was applied with * P < 0.0001. FL, fluorescence. c-f Red blood cell (RBC), platelet, monocyte and lymphocyte counts in peripheral blood in mice treated with control antibody or anti-Ly6G antibody at 14 days after stroke (n = 3). g Representative H&E stained sections at 14 days after stroke from mice treated with control antibody or anti-Ly6G antibody (n = 6). h Quantification of the infarct volume (n = 6). Statistical analysis was performed using unpaired two-tailed Student’s t test. Data are presented as mean ± SD. Source data underlying graph b, c, d, e, f, h are provided as a Source Data file.

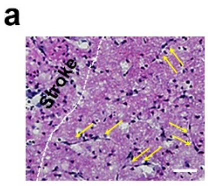

Supplementary Figure 3. a, H&E-stained sections showing the presence of extracellular DNA (arrows) in the peri-infarct cortex at 3 days after stroke. Bar = 40 μm**.** Independent experiments are repeated at least three times.

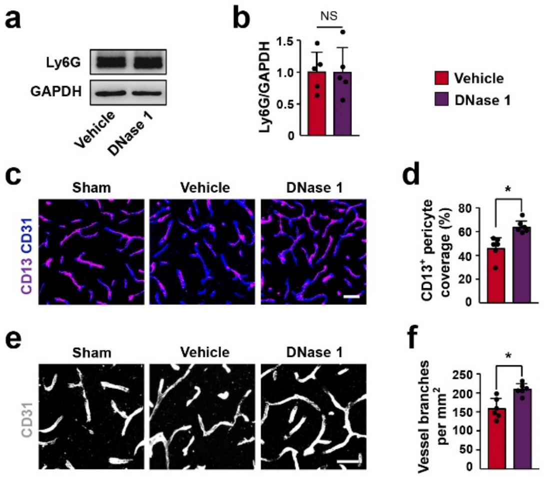

Supplementary Figure 4. Treatment with DNase 1 enhanced CD13^{+} pericyte coverage and vascular branches, whereas did not affect neutrophil recruitment. a, b Western blot analysis of the amount of neutrophils at 3 days after stroke in mice treated with vehicle or

DNase 1 (n = 5), unpaired two-tailed Student's t test was applied with P = 0.9878. c, d Representative images (c) and quantification (d) of CD13-positive pericyte coverage on CD31-positive brain capillaries at 14 days after stroke in mice treated with vehicle or DNase 1 (n = 6), unpaired two-tailed Student's t test was applied with * P = 0.0022. Bar = 40 μm. e, f Representative images (e) and quantification (f) of vascular branches at 14 days after stroke in mice treated with vehicle or DNase 1 (n = 6), unpaired two-tailed Student's t test was applied with * P = 0.0024. Bar = 40 μm. Data are presented as mean ± SD. Source data underlying graph a, b, d, f are provided as a Source Data file.

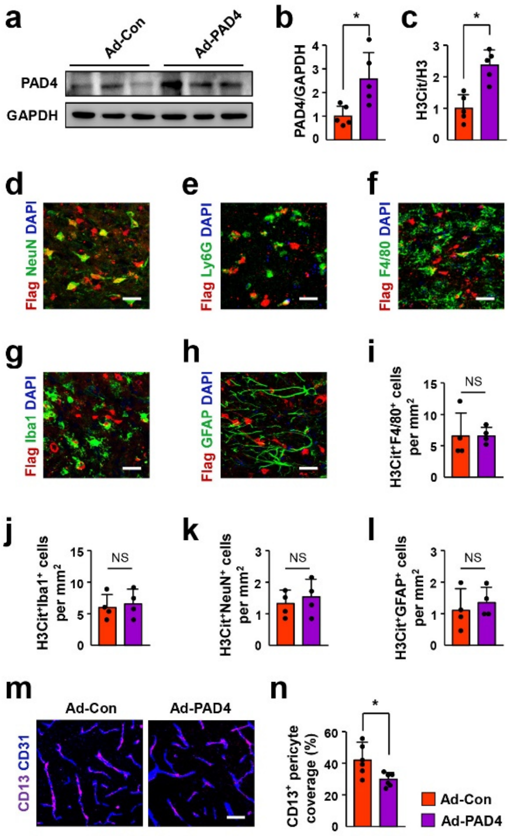

Supplementary Figure 5. The efficiency of PAD4 expression and cellular distribution after injection of recombinant PAD4 adenovirus. a, b Western blot confirmed that injection of PAD4 adenovirus successfully induced upregulation of PAD4 protein expression in the ischemic cortex at 3 days (n = 5 biologically independent experiments), unpaired two-tailed Student's t test was applied with * P = 0.0186. c Quantification of

H3Cit levels in the ischemic cortex at 3 days in mice treated with control or PAD4 adenovirus (n = 5), unpaired two-tailed Student's t test was applied with * P = 0.0015. d-h PAD4-flag was present in neurons, neutrophils, macrophages/microglial cells and microglial cells at 3 days after stroke, but was rarely detected in astrocytes. Bar = 20 μm. Independent experiments are repeated at least three times. i-l PAD4 adenoviruses treatment did not affect the number of H3Cit$^+$ macrophages/microglial cells, H3Cit$^+$ microglial cells, H3Cit$^+$ neurons, and H3Cit$^+$ astrocytes at 3 days after stroke (n = 4), unpaired two-tailed Student's t test was applied with P = 0.9915 (i), P = 0.7386 (j), P = 0.5838 (k), P = 0.5771(l). m, n Representative images (m) and quantitative analysis (n) of CD13-positive pericyte coverage on CD31-positive brain capillaries at 14 days after stroke in mice treated with control or PAD4 adenovirus (n = 6), unpaired two-tailed Student's t test was applied with * P = 0.0172. Bar = 40 μm. Data are presented as mean ± SD. Source data underlying graph a, b, c, i, j, k, l, n are provided as a Source Data file.

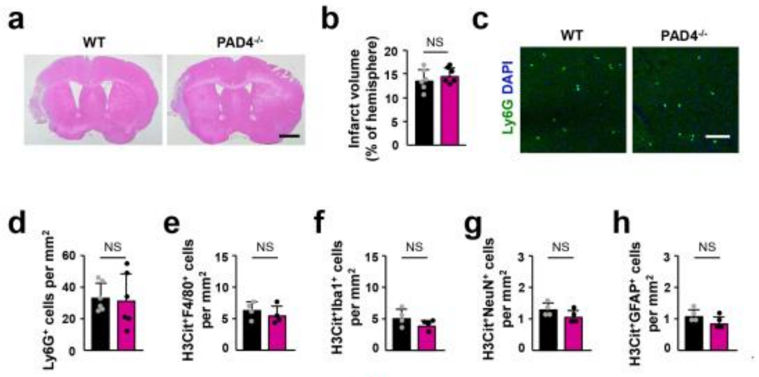

Supplementary Figure 6. Effects of PAD4 deficiency on infarct volumes, the number of neutrophils, H3Cit$^+$ macrophages/microglial cells, H3Cit$^+$ microglial cells, H3Cit$^+$ neurons, and H3Cit$^+$ astrocytes. a Representative H&E stained sections at 14 days after stroke from WT and PAD4$^{-/-}$ mice (n = 6). b Quantification of the infarct volume (n = 6). c, d Representative images (c) and quantification (d) of Ly6G-positive neutrophils in the peri-infarct cortex at 14 days in WT and PAD4$^{-/-}$ mice (n = 6). Bar = 40 μm. e-h

Quantification of the number of H3Cit⁺ macrophages/microglial cells (e), H3Cit⁺ microglial cells (f), H3Cit⁺ neurons (g), and H3Cit⁺ astrocytes (h) at 3 days after stroke in WT and PAD4$^{-/-}$ mice (n = 4). Statistical analysis was performed using unpaired two-tailed Student's t test. Data are presented as mean ± SD. Source data underlying graph b, d, e, f, g, h are provided as a Source Data file.

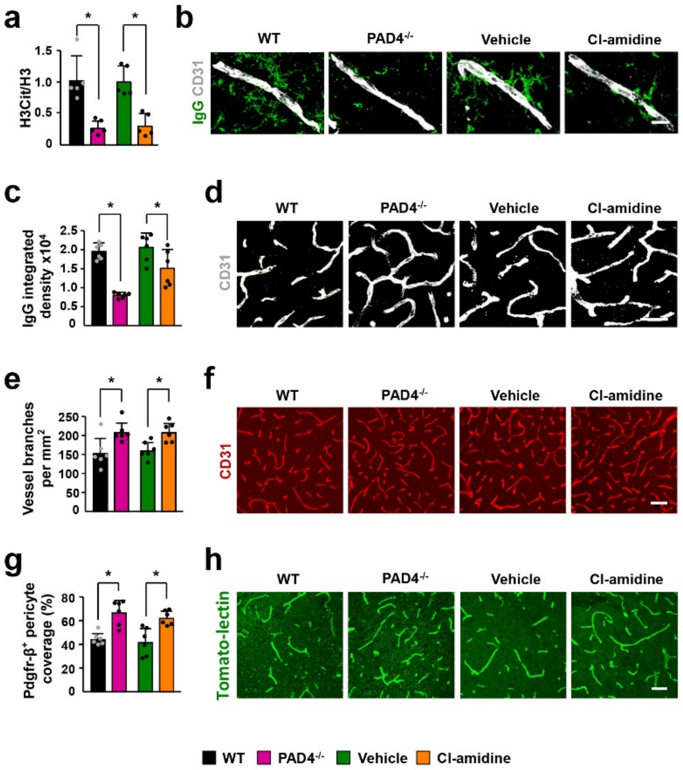

Supplementary Figure 7. PAD4 deficiency or inhibition by Cl-amidine reduced NETs and BBB breakdown, and promotes neovascularization. a Quantification of H3Cit levels in the peri-infarct cortex at 3 days in WT and PAD4$^{-/-}$ mice, and WT mice treated with vehicle or the PAD inhibitor Cl-amidine (n = 5), Mann-Whitney test was applied with * P = 0.0079 (WT vs PAD4$^{-/-}$), * P = 0.0079 (Vehicle vs Cl-amidine). Data show results from three technical replicates. b, c Representative images of IgG deposits (b) and quantification of extravascular IgG deposits (c) at 14 days after stroke (n = 6), Mann-Whitney test was applied with * P = 0.0022 (WT vs PAD4$^{-/-}$), unpaired two-tailed Student's t test was applied with * P = 0.0463 (Vehicle vs Cl-amidine). Bar = 20 µm. d, e

Representative images (d) and quantification (e) of vascular branches at 14 days after stroke (n = 6), unpaired two-tailed Student's t test was applied with * P = 0.0210 (WT vs PAD4$^{-/-}$), * P = 0.0068 (Vehicle vs Cl-amidine). Bar = 40 μm. f Representative images of CD31-positive microvessels in the peri-infarct cortex at 14 days for each group. g Quantification of pericyte coverage in the peri-infarct cortex (n = 6), unpaired two-tailed Student's t test was applied with * P = 0.0009 (WT vs PAD4$^{-/-}$), * P = 0.0040 (Vehicle vs Cl-amidine). h Representative images of tomato-lectin perfused vessels in the peri-infarct cortex at 14 days. Bar = 40 μm. Data are presented as mean ± SD. Source data underlying graph a, c, e, g are provided as a Source Data file.

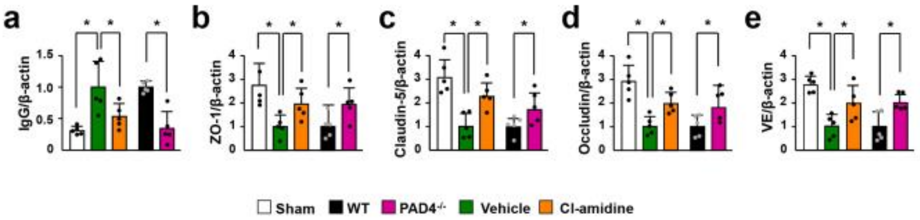

Supplementary Figure 8. PAD4 deficiency or inhibition by Cl-amidine reduced BBB breakdown and preserved BBB integrity. a-e Immunoblot quantification of IgG levels in capillary-depletion brain tissue (a), the tight junction protein ZO-1 (b), claudin-5 (c), occludin (d) and the adherens junction protein VE-cadherin (e) in isolated brain microvessels at 14 days after stroke (n = 5). One-way ANOVA test was applied with * P = 0.0032 (Sham vs Vehicle from a), * P = 0.0376 (Vehicle vs Cl-amidine from a), * P = 0.0056 (Sham vs Vehicle from b), * P = 0.0323 (Vehicle vs Cl-amidine from b), * P = 0.0006 (Sham vs Vehicle from c), * P = 0.0185 (Vehicle vs Cl-amidine from c), * P = 0.0003 (Sham vs Vehicle from d), * P = 0.0317 (Vehicle vs Cl-amidine from d), * P = 0.0003 (Sham vs Vehicle from e), * P = 0.0373 (Vehicle vs Cl-amidine from e), unpaired two-tailed Student's t test was applied with * P = 0.0008 (WT vs PAD4$^{-/-}$ from a), * P = 0.0393 (WT vs PAD4$^{-/-}$ from b), * P = 0.0242 (WT vs PAD4$^{-/-}$ from c), * P = 0.0322

(WT vs PAD4$^{-/-}$ from d), Mann-Whitney test was applied with * P = 0.0153 (WT vs PAD4$^{-/-}$ from e). Data are presented as mean ± SD. Source data underlying graph a, b, c, d, e are provided as a Source Data file

.

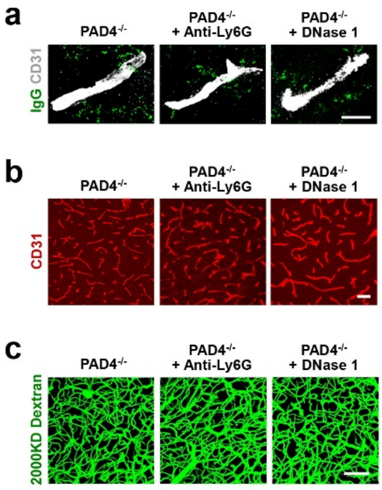

Supplementary Figure 9. a-c Representative images of IgG extravascular deposits (a), CD31-positive microvessels (b) and perfused cortical capillaries with intravenously injected FITC-dextran (c) in the peri-infarct cortex at 14 days in PAD4$^{-/-}$ mice treated with either vehicle, anti-Ly6G antibody or DNase 1. Bar = 10μm (a), 40μm (b) and 100 μm (c). Independent experiments are repeated at least three times.

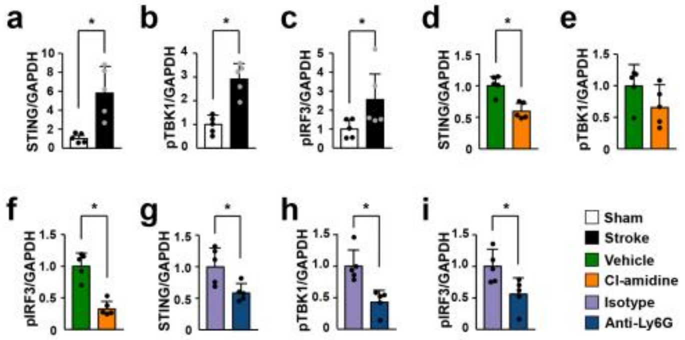

Supplementary Figure 10. Neutrophil depletion and the PAD inhibitor Cl-amidine reduced stroke-induced upregulation of STING and activation of TBK1 and IRF3. a-c Quantification of STING (a), phosphorylated TBK1 (p-TBK1) (b) and p-IRF3 (c) in the

cortex of mice without stroke or at day 3 after stroke (n = 5), Mann-Whitney test was applied with * P = 0.0079 (a), * P = 0.0278 (c), unpaired two-tailed Student's t test was applied with * P = 0.0014 (b). d-i Treatment of mice with Cl-amidine (d-f) or anti-Ly6G antibody (g-i) decreased the levels of STING (d, g), p-TBK1 (e, h) and p-IRF3 (f, i) in the cortex at day 3 after stroke (n =5), unpaired two-tailed Student's t test was applied with * P = 0.0014 (d), * P = 0.0002 (f), * P = 0.0108 (g), * P = 0.0040 (h) , * P = 0.0215 (i), and Mann-Whitney test was applied with P = 0.0952 (e). Data are presented as mean ± SD. Source data underlying graph a, b, c, d, e, f, g, h, i are provided as a Source Data file.

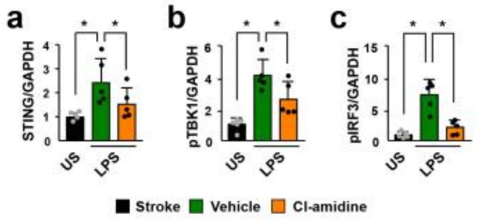

Supplementary Figure 11. a-c The PAD inhibitor Cl-amidine reduced LPS-induced upregulation of STING and activation of TBK1 and IRF3 in isolated neutrophils from mice at 3 days after stroke (n = 5). One-way ANOVA test was applied with * P = 0.0118 (Stroke vs Vehicle from a), * P = 0.0003 (Stroke vs Vehicle from b), * P = 0.0451 (Vehicle VS Cl-amidine from b), * P < 0.0001 (Stroke vs Vehicle from c), * P = 0.0004 (Vehicle VS Cl-amidine from c). Data are presented as mean ± SD. Source data underlying graph a, b, c are provided as a Source Data file.
