# Supplementary material for: Neutrophil extracellular traps released by neutrophils impair revascularization and vascular remodeling after stroke
Source: Nat Commun. 2020 May 19;11:2488. doi: 10.1038/s41467-020-16191-y (PMC7237502; doi:10.1038/s41467-020-16191-y)
Supplement: Supplementary file 3 — Reporting Summary [file 41467_2020_16191_MOESM3_ESM.pdf]

nature research

Corresponding author(s): Bing-Qiao Zhao

Last updated by author(s): Mar 16, 2020

Reporting Summary

Nature Research wishes to improve the reproducibility of the work that we publish. This form provides structure for consistency and transparency in reporting. For further information on Nature Research policies, see Authors & Referees and the Editorial Policy Checklist.

Statistics

For all statistical analyses, confirm that the following items are present in the figure legend, table legend, main text, or Methods section.

n/a	Confirmed	
☐	☒	The exact sample size (*n*) for each experimental group/condition, given as a discrete number and unit of measurement
☐	☒	A statement on whether measurements were taken from distinct samples or whether the same sample was measured repeatedly
☐	☒	The statistical test(s) used AND whether they are one- or two-sided<br>*Only common tests should be described solely by name; describe more complex techniques in the Methods section.*
☒	☐	A description of all covariates tested
☐	☒	A description of any assumptions or corrections, such as tests of normality and adjustment for multiple comparisons
☐	☒	A full description of the statistical parameters including central tendency (e.g. means) or other basic estimates (e.g. regression coefficient) AND variation (e.g. standard deviation) or associated estimates of uncertainty (e.g. confidence intervals)
☐	☒	For null hypothesis testing, the test statistic (e.g. *F*, *t*, *r*) with confidence intervals, effect sizes, degrees of freedom and *P* value noted<br>*Give P values as exact values whenever suitable.*
☒	☐	For Bayesian analysis, information on the choice of priors and Markov chain Monte Carlo settings
☒	☐	For hierarchical and complex designs, identification of the appropriate level for tests and full reporting of outcomes
☒	☐	Estimates of effect sizes (e.g. Cohen's *d*, Pearson's *r*), indicating how they were calculated

Our web collection on statistics for biologists contains articles on many of the points above.

Software and code

Policy information about availability of computer code

Data collection	Immunohistochemical and TPLSM data were analyzed using ImageJ 1.46r software, Flow cytometry data were analyzed using FlowJo V10 software, Western blot data were analyzed using Image Lab-5.2.1. In vivo multiphoton microscopy data were analyzed using Olympus FV 10-ASW 4.2 Viewer software.
Data analysis	GraphPad Prism 7, FlowJo V10, ImageJ 1.46r, Image Lab-5.2.1 were used for statistical analysis.

For manuscripts utilizing custom algorithms or software that are central to the research but not yet described in published literature, software must be made available to editors/reviewers. We strongly encourage code deposition in a community repository (e.g. GitHub). See the Nature Research guidelines for submitting code & software for further information.

Data

Policy information about availability of data

All manuscripts must include a data availability statement. This statement should provide the following information, where applicable:

- Accession codes, unique identifiers, or web links for publicly available datasets
- A list of figures that have associated raw data
- A description of any restrictions on data availability

All data supporting the findings of this study are available within the article and its supplementary information files or from the corresponding author upon reasonable request.

Field-specific reporting

Please select the one below that is the best fit for your research. If you are not sure, read the appropriate sections before making your selection.

☒ Life sciences ☐ Behavioural & social sciences ☐ Ecological, evolutionary & environmental sciences

For a reference copy of the document with all sections, see nature.com/documents/nr-reporting-summary-flat.pdf

Life sciences study design

All studies must disclose on these points even when the disclosure is negative.

Sample size	Sample size was indicated in each figure legend and determined acccording to previous experiments from our laboratory and others. For example, Xu H et al. ADAMTS13 controls vascular remodeling by modifying VWF reactivity during stroke recovery[J]. Blood, 2017:blood-2016-10-747089. Experiments conducted with the minimum required number per groups would be necessary to obtain significantly different results to support meaningful conclusions.
Data exclusions	No data were excluded from the analyses.
Replication	All experiments were performed independently at least three times using biologically independent replicates, the number of replicates are mentioned in the figure legends section. All replication attempts were successful.
Randomization	Mice were randomly assigned to groups.
Blinding	Behavioral tests were performed by an investigator blinded to the experimental groups

Reporting for specific materials, systems and methods

We require information from authors about some types of materials, experimental systems and methods used in many studies. Here, indicate whether each material, system or method listed is relevant to your study. If you are not sure if a list item applies to your research, read the appropriate section before selecting a response.

Materials & experimental systems

n/a	Involved in the study	
☐	☒	Antibodies
☒	☐	Eukaryotic cell lines
☒	☐	Palaeontology
☐	☒	Animals and other organisms
☒	☐	Human research participants
☒	☐	Clinical data

Methods

n/a	Involved in the study	
☒	☐	ChIP-seq
☐	☒	Flow cytometry
☒	☐	MRI-based neuroimaging

Antibodies

Antibodies used

Western Blot
rabbit anti-Histone H3 (anti-H3; 1:1000, 9715), rabbit anti-TANK-binding kinase 1 (pTBK1; 1:1000, 3504), rabbit anti-pTBK1 (1:1000, 5483), rabbit anti-interferon regulatory factor 3 (IRF3; 1:1000, 4302), rabbit anti-pIRF3 (1:1000, 4947), rabbit anti-β-actin (1:2000, 4970), rabbit anti-reduced glyceraldehyde-phosphate dehydrogenase (GAPDH; 1:2000, 5174, all from Cell Signaling Technology, MA), rabbit anti-H3Cit (1:1000, ab5103), rabbit anti-CD144 (vascular endothelial cadherin, VE-cadherin; 1:1000, ab33168), rabbit anti-occludin (1:1000, ab167161), rabbit anti-claudin-5 (1:1000, ab15106. all from Abcam), rat anti-Ly6G (1:1000, 551459, BD Pharmingen), mouse anti-PAD4 (O94H5 clone; 1:1000, 684202, Biolegend), rabbit anti-Zonula occludens-1 (ZO-1; 1:1000, 617300, Invitrogen), sheep anti-stimulator of interferon genes (STING; 1:1000, AF6516, R&D Systems
Immunofluoresence
anti-Ly6G (1:200, 551459, BD Pharmingen), anti-CD31 (PECAM-1; 1:200, 550274, BD Pharmingen), anti-CD31 (1:200, AF3628, R&D Systems), anti-H3Cit (1:1000, ab5103, Abcam), FITC-conjugated rat anti-aminopeptidase N (CD13; 1:200, 558744, BD Pharmingen), anti-platelet-derived growth factor receptor beta (Pdgfr-β; 1:200, AF385, R&D Systems), Hoechst 33342 (1:10000, H3570, Invitrogen), rat anti-F4/80 (1:200, ab6640, Abcam), goat anti-Iba1 (1:200, ab5076, Abcam), goat anti-GFAP (1:200, ab53554, Abcam), mouse anti-neuronal nuclei (NeuN; 1:200, MAB377, Millipore), rabbit anti-NeuN (1:200, ab177487, Abcam), mouse anti-Flag (1:200, 8146, Cell Signaling Technology, MA), Alexa Fluor 594-conjugated donkey anti-rabbit IgG (1:1000, A-21207, Invitrogen), Alexa Fluor 594-conjugated donkey anti-goat IgG (1:1000, A-11058, Invitrogen), Alexa Fluor 488-conjugated donkey anti-rat IgG (1:1000, A-21208, Invitrogen), Alexa Fluor 488-conjugated donkey anti-mouse IgG (1:1000, A-21202, Invitrogen), Alexa Fluor 647-conjugated donkey anti-goat IgG (1:1000, A-21447, Invitrogen), biotin-donkey anti-rat IgG (1:1000, A18743, Invitrogen)
Flow cytometry
FITC-conjugated rat monoclonal anti-mouse neutrophil antibody [7/4] (1:300, ab53453, Abcam), anti-mouse CD16/32 Fc block (2.4G2 clone; 1:100, 553141, BD Pharmingen), APC-Cy7-conjugated antibody to CD11b (Integrin alpha M, M1/70 clone; 1:200,

557657, BD Pharmingen), V450-conjugated antibody to CD45 (30-F11 clone; 1:200, 560501, BD Pharmingen), PE-conjugated antibody to Ly6G (1A8 clone; 1:200, eBioscience)

Validation

The antibodies are from commercial sources. For validation, the following methods were used: 1) use of isotype controls for analysis, 2) results from previous publications from our lab, 3) manufacturer provided validation on the same species, relevant information on the antibodies are available on the manufacture's websites.
Validation details of the primary antibodies are available on the manufacturers' websites:
Histone H3 (anti-H3; 9715), https://www.cst-c.com.cn/products/primary-antibodies/histone-h3-antibody/9715;
Anti-TANK-binding kinase 1 (TBK1; 3504), https://www.cst-c.com.cn/products/primary-antibodies/tbk1-nak-d1b4-rabbit-mab/3504
anti-pTBK1 (5483), https://www.cst-c.com.cn/products/primary-antibodies/phospho-tbk1-nak-ser172-d52c2-xp-rabbit-mab/5483?site-search-type=Products;
anti-interferon regulatory factor 3 (IRF3; 4302), https://www.cst-c.com.cn/products/primary-antibodies/irf-3-d83b9-rabbit-mab/4302;
anti-pIRF3 (4947), https://www.cst-c.com.cn/products/primary-antibodies/phospho-irf-3-ser396-4d4g-rabbit-mab/4947;
anti-β-actin (4970), https://www.cst-c.com.cn/products/primary-antibodies/b-actin-13e5-rabbit-mab/4970;
anti-reduced glyceraldehyde-phosphate dehydrogenase (GAPDH; 5174), https://www.cst-c.com.cn/products/primary-antibodies/gapdh-d16h11-xp-rabbit-mab/5174;
anti-H3Cit (ab5103), https://www.abcam.com/histone-h3-citrulline-r2-r8-r17-antibody-chip-grade-ab5103.html;
VE-cadherin (ab33168), https://www.abcam.com/ve-cadherin-antibody-intercellular-junction-marker-ab33168.html;
anti-occludin (ab167161), anti-claudin-5 (ab15106), https://www.abcam.com/occludin-antibody-epr8208-ab167161.html;
anti-Ly6G (551459, BD Pharmingen), https://www.bdbiosciences.com/cn/reagents/research/antibodies-buffers/immunology-reagents/anti-mouse-antibodies/cell-surface-antigens/purified-rat-anti-mouse-ly-6g-1a8/p/551459;
anti-PAD4 (O94H5 clone; 684202, Biolegend), https://www.biolegend.com/en-us/products/purified-anti-padi4-antibody-13127;
anti-Zonula occludens-1 (ZO-1; 617300, Invitrogen), https://www.thermofisher.com/cn/zh/antibody/product/ZO-1-Antibody-Polyclonal/61-7300;
anti-stimulator of interferon genes (STING; AF6516, R&D Systems), https://www.rndsystems.com/cn/products/human-sting-tmem173-antibody_af6516;
anti-Ly6G (1:200, 551459, BD Pharmingen), https://www.bdbiosciences.com/cn/reagents/research/antibodies-buffers/immunology-reagents/anti-mouse-antibodies/cell-surface-antigens/purified-rat-anti-mouse-ly-6g-1a8/p/551459;
anti-CD31 (PECAM-1; 1:200, 550274, BD Pharmingen), https://www.bdbiosciences.com/cn/applications/research/stem-cell-research/cancer-research/mouse/purified-rat-anti-mouse-cd31-mec-133/p/550274;
anti-CD31 (1:200, AF3628, R&D Systems), https://www.rndsystems.com/cn/products/mouse-rat-cd31-pecam-1-antibody_af3628;
anti-H3Cit (1:1000, ab5103, Abcam), https://www.abcam.com/histone-h3-citrulline-r2-r8-r17-antibody-chip-grade-ab5103.html;
FITC-conjugated rat anti-aminopeptidase N (CD13; 1:200, 558744, BD Pharmingen), https://www.bdbiosciences.com/cn/applications/research/stem-cell-research/hematopoietic-stem-cell-markers/mouse/negative-markers/fitc-rat-anti-mouse-cd13-r3-242/p/558744;
Hoechst 33342 (1:10000, H3570, Invitrogen); https://www.thermofisher.com/order/catalog/product/H3570?SID=srch-hj-H3570#/H3570?SID=srch-hj-H3570
anti-platelet-derived growth factor receptor beta (Pdgfr-β; 1:200, AF385, R&D Systems), https://www.rndsystems.com/cn/products/human-pdgf-rbeta-antibody_af385;
rat anti-F4/80 (1:200, ab6640, Abcam), https://www.abcam.com/f480-antibody-cia3-1-macrophage-marker-ab6640.html;
goat anti-Iba1 (1:200, ab5076, Abcam), https://www.abcam.com/iba1-antibody-ab5076.html;
goat anti-GFAP (1:200, ab53554, Abcam), https://www.abcam.com/gfap-antibody-ab53554.html;
mouse anti-neuronal nuclei (NeuN; 1:200, MAB377, Millipore), https://www.sigmaaldrich.com/catalog/product/mm/mab377?lang=zh&region=CN;
rabbit anti-NeuN (1:200, ab177487, Abcam), https://www.abcam.com/neun-antibody-epr12763-neuronal-marker-ab177487.html;
mouse anti-Flag (1:200, 8146, Cell Signaling Technology, MA), https://www.cst-c.com.cn/products/primary-antibodies/dykddddk-tag-9a3-mouse-mab-binds-to-same-epitope-as-sigma-s-anti-flag-m2-antibody/8146.

Animals and other organisms

Policy information about studies involving animals; ARRIVE guidelines recommended for reporting animal research

Laboratory animals

Peptidylarginine deiminase 4-deficient (PAD4-/-) mice on a C57BL/6J background were purchased from The Jackson Laboratory. Age-matched wild-type (WT) C57BL/6 mice were purchased from SLAC Laboratory Animal Co. Ltd., Shanghai, China.
Male mice (8-10 weeks, 23-26 g) kept in cages in standard housing conditions (temperature, 22℃± 2℃; humidity, 55% ± 5%; 12-hour light/ 12-hour dark cycle) were used in all animal experiments.

Wild animals

The study did not involve wild animals.

Field-collected samples

The study did not involve field-collected samples.

Ethics oversight

Experimental procedures performed on the mice were reviewed and approved by the Animal Care and Use Committee of the Institutes of Brain Science, Fudan University

Note that full information on the approval of the study protocol must also be provided in the manuscript.

Flow Cytometry

Plots

Confirm that:

☒ The axis labels state the marker and fluorochrome used (e.g. CD4-FITC).

☒ The axis scales are clearly visible. Include numbers along axes only for bottom left plot of group (a 'group' is an analysis of identical markers).

☒ All plots are contour plots with outliers or pseudocolor plots.

☐ A numerical value for number of cells or percentage (with statistics) is provided.

Methodology

Sample preparation	Peripheral blood was subjected to red blood cell lysis buffer (155 mmol/L NH4Cl, 10 mmol/L KHCO3, and 0.1 mmol/L Na2EDTA). Cells were washed with phosphate buffered saline (PBS) containing 1% bovine serum albumin (BSA) and resuspended in rat anti-mouse CD16/32 Fc block (2.4G2 clone; 1:100, 553141, BD Pharmingen) in PBS. Cell suspension was incubated with Allophycocyanin-cyanine dye (APC-Cy7)-conjugated antibody to CD11b (Integrin alpha M, M1/70 clone; 1:200, 557657, BD Pharmingen), V450-conjugated antibody to CD45 (30-F11 clone; 1:200, 560501, BD Pharmingen) and PE-conjugated antibody to Ly6G (1A8 clone; 1:200, eBioscience)
Instrument	BD LSRFortessaTM (BD Biosciences)
Software	FlowJo software (Tree Star Inc., Ashland, OR) and FlowJo V10 software
Cell population abundance	Neutrophils were confirmed to be >90%
Gating strategy	For gating , granulocytes were gated using forward scatter height (FSC-H) versus side scatter height(SSC-H), neutrophil surface marker positive events were gated using a FITC-conjugated rat monoclonal anti-mouse neutrophil antibody (1:300, anti-7/4, Abcam, cat. no. ab53453) versus fluorescence height

☒ Tick this box to confirm that a figure exemplifying the gating strategy is provided in the Supplementary Information.
